# Precision Population Cancer Medicine in Cancer of the Uterine Cervix: A Potential Roadmap to Eradicate Cervical Cancer

**DOI:** 10.7759/cureus.53733

**Published:** 2024-02-06

**Authors:** Mary R Nittala, Johnny Yang, Alexander E Velazquez, John D Salvemini, Gregory R Vance, Camille C Grady, Bradley Hathaway, Jeffrey A Roux, Srinivasan Vijayakumar

**Affiliations:** 1 Radiation Oncology, University of Mississippi Medical Center, Jackson, USA

**Keywords:** precision population medicine, uterine cervical cancer, cancer prevention, big-data, genomic medicine, precision medicine

## Abstract

With the success of the Human Genome Project, the era of genomic medicine (GM) was born. Later on, as GM made progress, there was a feeling of exhilaration that GM could help resolve many disease processes. It also led to the conviction that personalized medicine was possible, and a relatively synonymous word, precision medicine (PM), was coined. However, the influence of environmental factors and social determinants of diseases was only partially given their due importance in the definition of PM, although more recently, this has been recognized. With the rapid advances in GM, big data, data mining, wearable devices for health monitoring, telemedicine, etc., PM can be more easily extended to population-level health care in disease management, prevention, early screening, and so on.and the term precision population medicine (PPM) more aptly describes it. PPM’s potential in cancer care was posited earlier,and the current authors planned a series of cancer disease-specific follow-up articles. These papers are mainly aimed at helping emerging students in health sciences (medicine, pharmacy, nursing, dentistry, public health, population health), healthcare management (health-focused business administration, nonprofit administration, public institutional administration, etc.), and policy-making (e.g., political science), although not exclusively. This first disease-specific report focuses on the cancer of the uterine cervix (CC). It describes how recent breakthroughs can be leveraged as force multipliers to improve outcomes in CC - by improving early detection, better screening for CC, potential GM-based interventions during the stage of persistent Human papillomavirus (HPV) infection and treatment interventions - especially among the disadvantaged and resource-scarce populations. This work is a tiny step in our attempts to improve outcomes in CC and ultimately eradicate CC from the face of the earth.

## Introduction and background

Medicine is changing [1,2], health care is changing [3-5], Disease diagnostic methods are changing [6-8]. The educated population’s health awareness is changing [9,10], and new disease outbreaks are happening [11-14]. The populations are changing their lifestyles to more Westernized habits, thus changing the disease profile in those regions [14-16]. Unstoppable population growth and unfavorable population age-related distributions are being identified in different nations [17,18]. Although longevity has increased in almost all nations, the gain is not even [19-23]. In this review and perspective, we posit that by using genomic medicine (GM), big data analysis, artificial intelligence, wearable devices, and other digital technological innovations defined as precision population medicine (PPM) [8], cancer care outcomes can be improved. In this communication, further emphasis is placed on the issues related to the disadvantaged population.

Healthcare leaders, including physician bodies, public health specialists, governing entities, political leaders, and people at large, understand the issues mentioned above and would like holistic solutions. In a series of reports, the current authors focus on improving cancer care outcomes and making such improvements more universal without excluding resource-scarce populations. This task is enormous. One small group can address only some of the issues. So, the current series of papers aims to show how precision population cancer medicine (PPCM) and PPM, as defined by us previously [8] can make a difference. Even in this small step to address the vast challenges, we aim to inform and educate students about healthcare improvement - medicine, nursing, pharmacy, health administration, public health, population health, political science, and so on [24]. Due to the goal of our inclusivity of a wide range of students who will make considerable contributions to the future health improvement and disease management of the future human race [25], certain portions of the narrative in these reports may sound simplistic and too basic to some - the authors seek their indulgence. The current report will focus on cancer of the uterine cervix.

**Figure 1:**
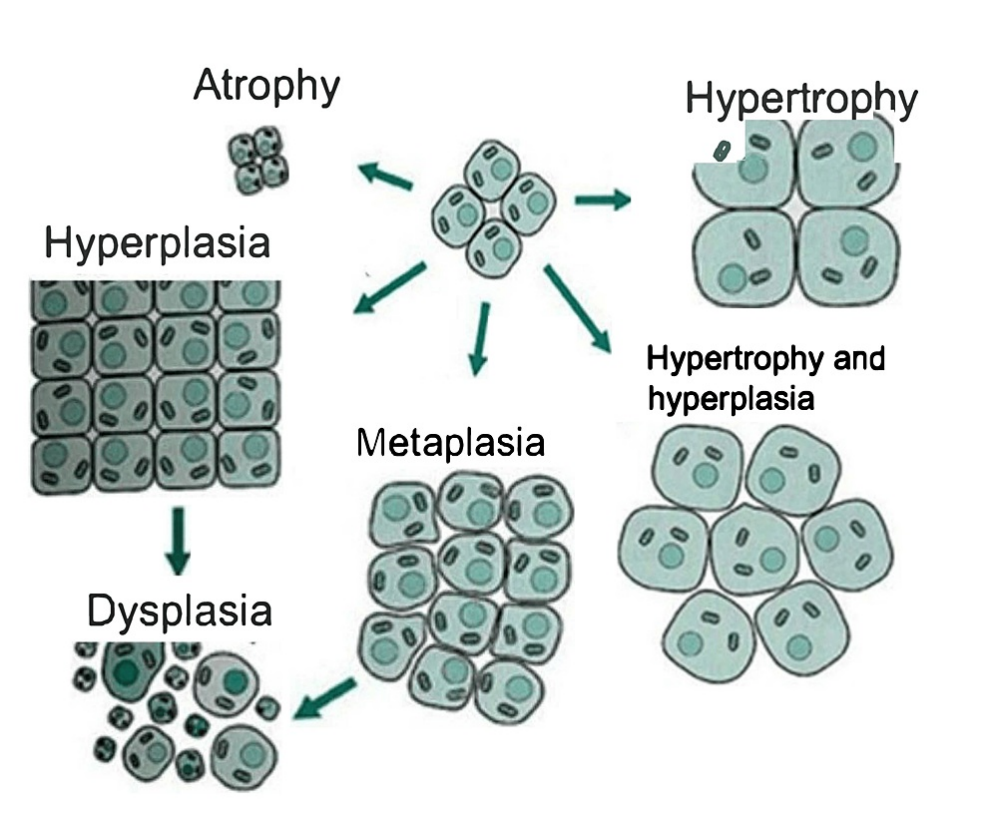
Evolution of cancer. This image is reproduced from sphweb [27], and permission was obtained from the licensed content publisher Boston University School of Public Health.

Cancer arises from the breakdown of normal cellular reproductive processes, in which the biological controls and checkpoints go awry or are bypassed completely. As a result, these cancerous cells grow independently and ignore signals meant to regulate cell growth and proliferation. As these cell numbers continue to increase, they progress into either a benign tumor that does not invade nearby tissues or a malignant tumor that does [26]. Eventually, the abnormal cells in a malignant mass can spread throughout the body and invade other tissues, leading to the formation of new tumors in a distant location of the body, otherwise known as metastasis. This mobility and malignancy arise from genes mutated to preferentially turn on or off to enhance growth, lifespan, energy usage, and cell blood flow [26]. Cancer must overcome many biological checkpoints, so the cells transform by accumulating these mutations. Atrophy occurs when normal cells reduce in cell size because of muscular dystrophy, and hypertrophy occurs due to the abnormal increase in cell size [27]. Figure 1 represents the transition of normally functioning cells to metaplasia (in which cell types are replaced by those not typically found in the specific tissue), which is the first step in the transformation process of cancerous growth [27].

Next, metaplasia may progress into dysplasia, a process where cells begin showing abnormal cellular architecture, size, number, and function [28]. The final and irreversible step in cell cancer transformation is neoplasia. The mutated cells become self-governing and are independent of all regulated physiological and biological signals, forming a rapidly expanding, completely autonomous lesion implicated in the evolution towards malignancy [29].

**Figure 2:**
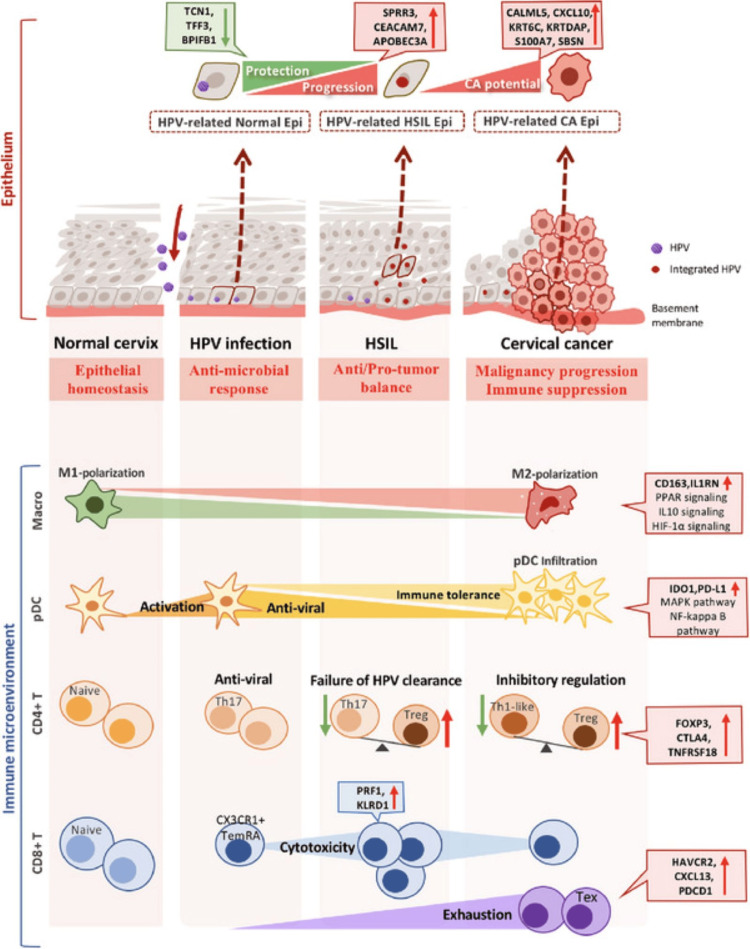
HPV-induced malignant transition in the epithelium and immune microenvironment. This image is reproduced from Guo et al. [33], and available via Creative Commons Attribution 4.0 International License. HPV, human papillomavirus; HSIL, high-grade squamous intraepithelial lesions; Epi, epithelial; CA, cancer antigen; TCN1, transcobalamin; TFF3, trefoil; BPIFB1, bactericidal/permeability-increasing-fold-containing family B member 1; SPRR3, small proline-rich protein gene; CEACAM7, carcinoembryonic antigen-related cell adhesion molecule 7; APOBEC3A, apolipoprotein B mRNA-editing enzyme catalytic 3A; CALML5, calmodulin-like protein gene; CXCL10, chemokine-interferon-y inducible protein 10 kDa; KRT6C, keratin type II cy cytoskeletal 6C gene; KRTDAP, keratinocyte differentiation associated protein; S100A7, S 100 calcium binding protein A7; SBSN,  subrabasin gene; CD163, cysteine-rich scavenger receptor; IL1RN, interleukin 1 receptor antagonist; PPAR, peroxisome proliferator-activated receptor; IL10, interleukin-10 gene; HIF-1, hypoxia-inducible factor-1; pDCs, plasmacytoid dendritic cells; CD4+T, clusters of differentiation 4 T helper cells; CD8+T, clusters of differentiation 8 T helper cells; IDO1, indoleamine-2,3-dioxygenase enzyme; PD-L1, programmed cell death ligand-1; MAPK, mitogen-activated protein kinase; NF-kappa B, nuclear factor-kB; Th17, T helper 17 cells; Treg, regulatory T cells; Th1-like, T helper cells 1; FOXP3, forkhead box P3 protein; CTLA4, cytotoxic T-lymphocyte associated antigen; TNFRSF18, tumor necrosis factor receptor superfamily member 18; CX3CR+, chemokine cytokine receptor; TemRA, tumor-specific CD8+ tumor-infiltrating lymphocytes; PRF1, perforin-1 gene; KLRD1, killer cell lectin like receptor D1; HAVCR2, hepatitis A virus cellular receptor 2; CXCL13, chemokine C-X-C motif ligand 13; PDCD1, programmed cell death 1 gene.

Cervical cancer (CC) is cancer of the cervix that is rapidly becoming one of the leading cancers in women in both incidence and frequency [30]. CC has two main subtypes: squamous cell carcinoma originates generally from the ectocervix, and adenocarcinoma develops from the endocervix [31]. A pertinent, almost inevitable initiating risk factor for this disease is a chronic Human papillomavirus (HPV) infection [30], particularly high-risk HPV subtypes, HPV 16, 18, 31, 33, present in 85% of all CC. This chronic infection allows genomic instability and uncontrolled cell proliferation, which may progress to malignancy [32]. Cervical carcinogenesis includes HPV infection, persistence, progression to the high-grade precancer lesion, and invasion. Figure 2 demonstrates the mechanism of malignancy HPV uses to transform the epithelium and microenvironment into a suitable habitat for cancer [33].

Even with CC being the fourth most common female cancer worldwide [32], with an incidence of 604,127 in 2019 and 341,831 deaths [34], the standard of care for this disease needs improvement. Table 1 represents the CC incidence and mortality rates in the United States and Mississippi [35,36]; Mississippi is included, being the state of the authors as well as a good example of regions with higher than average incidence and mortality.

**Table 1:** Cervical cancer incidence and mortality rates in the United States and Mississippi [35,36]. This information from CDC and MS cancer registry are in the public domain and can be freely used or reproduced without obtaining copyright permission.

Variable	United States (2015-2019)		Mississippi (2015-2020)	
Type of race	Incidence	Mortality	Incidence	Mortality
Caucasians	38,734	12,940	505	200
African-Americans	9,645	3,813	329	147
Other races	16,619	4,107	14	5
Total	64,998	20,860	848	352

Given that treatments following diagnosis are stage-dependent and other prognostic factors, CC care can vary with several health outcomes. However, if diagnosed earlier, CC has a five-year survival of over 90%. In comparison, the five-year survival rate of CC when diagnosed at an advanced, for example, distant the metastatic stage, is reduced to 20% [32]. The standard of care for early-stage, localized CC includes surgery, chemotherapy, and radiation therapy. Care for metastasized CC includes treatments focusing on systemic approaches, such as chemotherapy, and more recently, the use of targeted agents and immunotherapies [32]. Given that the prognosis of the disease is invariably poor for later staged diagnoses, CC treatment must evolve and progress into more effective strategies to yield improved patient outcomes.

PM tailors the treatment to the individual rather than just the stage of the disease. PM focuses on the specificity of the patient’s genes, environment, and lifestyle to personalize the treatment, aiming for improved outcomes [8]. To take patient care a step further, we will incorporate population-level assessment, screening, and prevention through the lens of PPM to create a comprehensive view of CC. PPM utilizes a larger-scale approach than PM by bringing in population data, epigenetics, and telehealth methods, which were not previously appreciated in the original PM. PPM will be able to assess high-risk individuals in the population, detect cancers earlier, and be utilized to create more effective treatment plans [8]. In support of using PPM, genomics, the “cornerstone of precision medicine studies,” has been used successfully to treat patients, for example, by matching drugs to the patients’ genomes to produce improved responsiveness to the medication.

Another example is personalized vaccines utilizing genome mapping to generate cancer-specific neo-epitopes [37]. In other words, vaccines are being delivered to recognize and target specific mutations in an individual cancer cell that the body recognizes as foreign. Genomics and precision medicine (PM) have recently been successfully used in managing many cancers, concluding that different variations of PM will help treat CC. PPM has been utilized in the management of CC successfully. For example, in one study, urine samples were processed for potential biomarkers for screening and early detection of CC [38], and in another study implementing PPM, a research team used a community component to their study, assessing the effectiveness of community-driven research in CC. The study found statistically significant effects of improving outcomes after the community’s inclusion in CC screening [39], highlighting the positive impact when employing PPM.

This paper details a few examples of the successful applications of PPM in CC to demonstrate the initial successes of such approaches and the future promise of PPM’s ability to improve outcomes in CC and benefit disadvantaged populations more efficiently and effectively. With decreasing resources worldwide, PPM’s strategies can deliver more value-efficient CC screening, prevention, and management.

## Review

Search databases and search strategy

This is a narrative review where databases such as MEDLINE/PubMed and Google Scholar were used for the literature search between of 1989 and 2023. The databases were screened with the individual medical subject heading keywords and key term combinations, including “precision population medicine,” “precision medicine,” “cervical cancer,” “HPV,” “epigenetics,” “big data,” “public health,” “telehealth,” “multi-cancer early detection,” and “cervical cancer detection.”

Inclusion/exclusion criteria

Studies only in English from 1989 to 2023 were included, with no other specific filters being used. Since we included only English language literature, there is likely to be a relatively limited genetic pool and bias to specific geographical regions. Commentaries, letters to the editor, and unpublished reports were excluded.

Genetic and molecular processes involved in the development of CC associated with HPV

Role of HPV in CC

HPV is a pervasive infection accounting for over 95% of CC cases that can be transmitted unknowingly due to its asymptomatic nature and prolonged incubation period [40]. This virus spreads through skin-to-skin contact (HPV 1,4) and is often sexually transmitted (HPV 16,18, 31, 33) [41]. There are over 130 known types of HPV, and 20 of these types have been linked to cancer [42]. Among the various types of HPV, 16 and 18 are the most frequently detected in cases of invasive CC. Continuous expression of E6 and E7, two transforming proteins encoded by high-risk HPVs such as 16 and 18, is necessary for the proliferation and survival of CC cell lines [43]. E6 and E7 exhibit carcinogenic effects by interacting with the tumor suppressor proteins p53 and Rb [43]. Analyzing the effects of E6 and E7 on these tumor suppressor proteins will provide an understanding of the pathogenesis of HPV-induced cancer.

Key Factors That Lead to Persistent HPV Infections and CC

Due to its widespread prevalence, tremendous progress has been made in better understanding the prevention and treatment of CC. Although not solely responsible, 99.7% of CC cases are caused by persistent genital infection of high-risk HPV [44]; CC arises from a complex interplay between inherited genetic predisposition and environmental factors. HPV infections alone are insufficient to cause cancer, as 60% of HPV infections regress within one year and 90% regress within two years; therefore, efforts to identify inherited genetic risk factors are vital to understanding the interactions between the host and virus [45]. Additionally, HPV-driven CC is rare since the host immune system eliminates most infections, and it takes several decades for persistent HPV infections to lead to CC. This extended time frame offers clinical intervention opportunities, such as identifying the carcinogenic pattern and key targets in the HPV-host interaction [45]. Previous research focused on variants’ potential roles in the cellular cycle, apoptosis, proliferation and differentiation, DNA repair, and immune responses [45]. Mechanistic links to CC progression is lacking, and genome-wide association studies are needed to understand the potential mechanisms of genetic vulnerability to CC. This section will focus on two oncogenes involved in persistent HPV infection, E6 and E7, that affect cellular pathways responsible for managing cell cycle control via interplay with p53 and pRb, two tumor-suppressing genes [43,46].

HPV E6 and E7: Targets of Detection and Therapy

**Figure 3:**
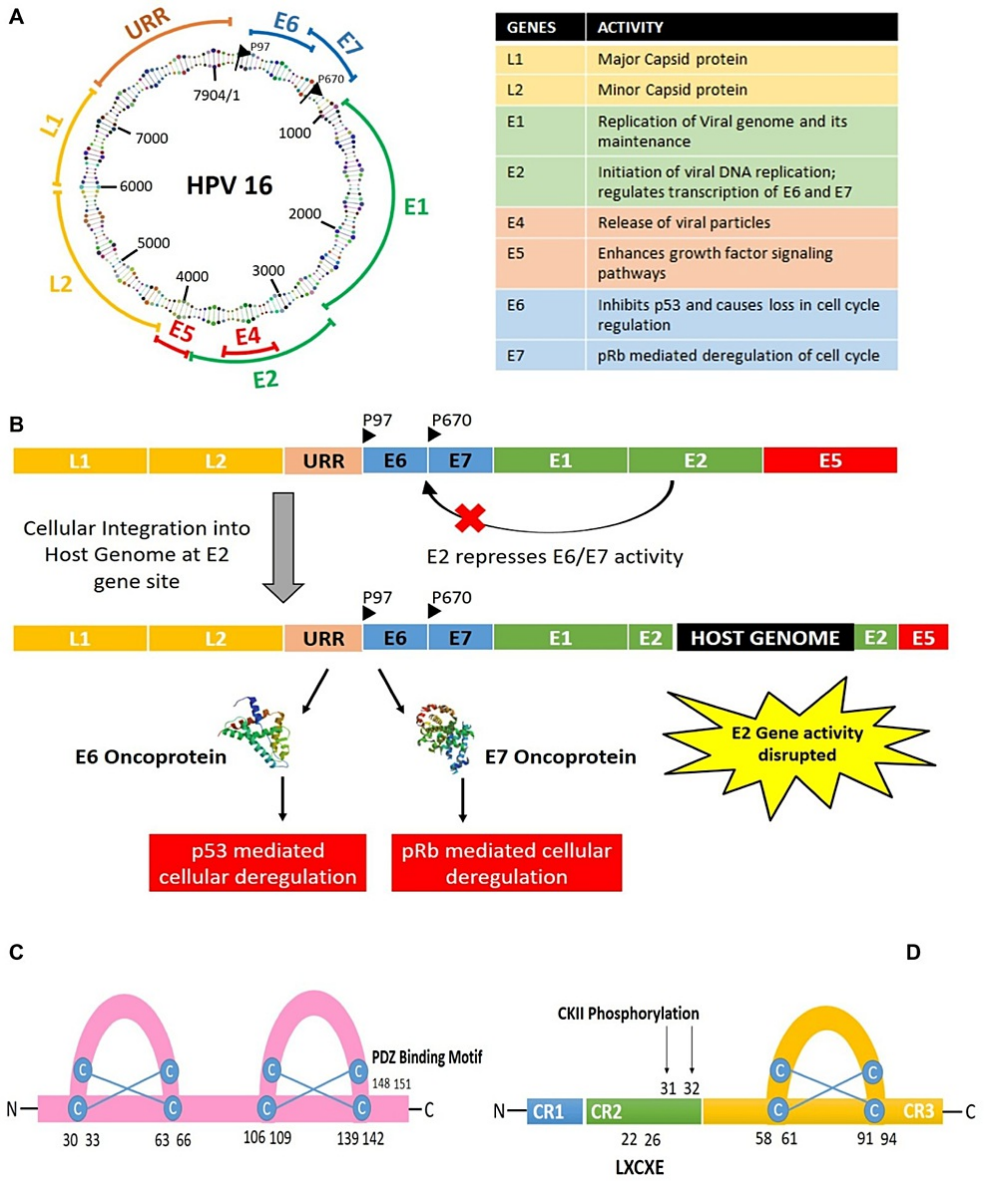
The carcinogenic process between HPV and the host. (A) Structure and organization of HPV16 genome. (B) Integration of HPV genome into the host genome via disruption of the E2 gene leading to the expression of the oncogenes E6 and E7. (C) Structure of E6 oncoprotein. (D) Structure of E7 oncoprotein. This image is reproduced from Pal et al. [43] and is available via Creative Commons Attribution 4.0 International License. HPV, human papillomavirus; URR, upstream regulatory region; p97, HPV-16 early promoter; p670, HPV-16 late promoter; p53, tumor suppressor gene; pRb, tumor suppressor gene retinoblastoma; CKII, high protein kinase; LXCXE, E7 domain.

The insertion of HPV DNA into the human genome is necessary for developing CC [47]. The primary establishment and progression of CC rely on two essential oncogenes, E6 and E7. When persistently expressed, these oncogenes result in tumorigenesis [43,46]. The synergistic actions of E6 and E7 facilitate the development of HPV-induced cancer by targeting various cellular pathways responsible for regulating cell cycle control through interactions with p53 and pRb, two tumor-suppressing genes [43,46]. Targeting E6 and E7, the biomarkers responsible for driving CC progression through therapeutic approaches, are highly effective in selectively removing the abnormally proliferating malignant cells [43]. Cutting-edge genome editing techniques that inhibit the activity of E6 and E7 have demonstrated efficacy in reducing the population of CC cells infected with HPV [43]. The progression of CC depends on genetic susceptibility and environmental factors, which will be discussed in later sections. This section focuses on understanding the carcinogenic process and relevant targets during the interaction between HPV and the host (Figures 3A-3D).

Furthermore, this discussion explores the cutting-edge technologies and treatments available for cancer interventions. By leveraging specific screening techniques and treatments, clinical PM can be achieved, which allows for the anticipation, prevention, and timely management of patients.

How Does the Association of E6 With p53 Affect the Progression of Cancer?

**Figure 4:**
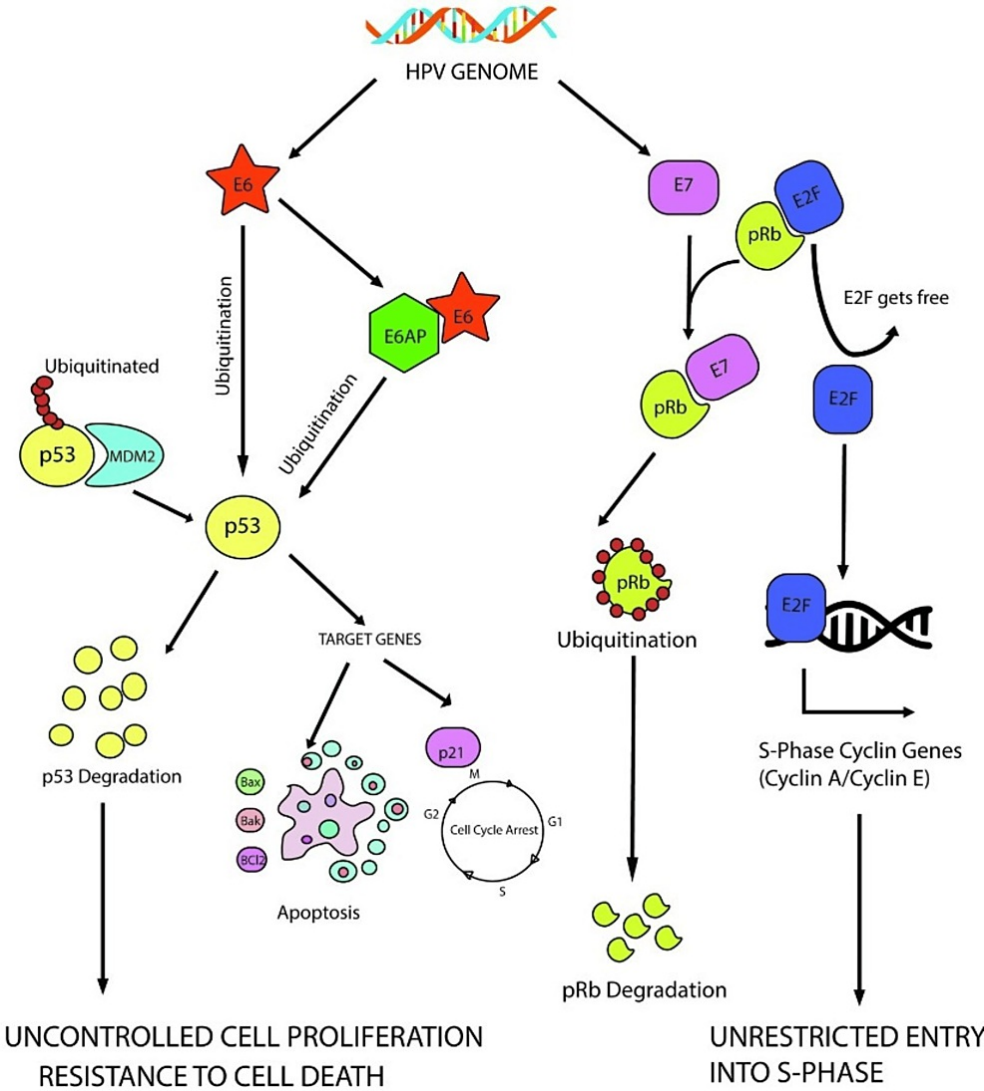
The role of HPV E6 and E7 oncogenic proteins in HPV-linked cervical cancer development. This image is reproduced from Pal et al. [43] and is available via Creative Commons Attribution 4.0 International License. HPV, human papillomavirus; E6, oncoprotein; E7, oncoprotein; E2F, prognostic biomarker; E6AP, E6 associated protein; pRb, tumor suppressor gene retinoblastoma; p53, cellular tumor suppressor protein; p21, inhibitor of the cell cycle; Bax, proapoptotic protein; Bak, proapoptotic protein; Bc12, antiapoptotic protein; G1, gap phase 1; G2, gap phase 2; M, mitosis; S, synthesis phase.

The p53 protein, as a transcription factor, regulates cell cycle progression and survival [48]. The regulatory role of p53 extends to the G1/S cell cycle checkpoint and the mitotic spindle checkpoint [46]. When exposed to DNA-damaging agents, p53 levels rise through a post-transcriptional mechanism. Stimulation of p53 results in elevated levels of p21 protein, which then induces cell cycle arrest by blocking cyclin-CDK activity [49]. Mutations in p53 are frequently observed in various human cancers, and the protein is believed to be involved in preserving genomic stability. However, the high-risk HPV E6 is known to impede all these functions of p53 [49]. When high-risk HPVs infect cells, the E6 oncogene interacts with E6-AP, an E3 ubiquitin ligase, to form a trimeric complex with p53 [49]. This leads to the degradation of p53 through the ubiquitin-proteasome pathway, causing a loss of both apoptotic functions and cell cycle control (Figure 4) [43].

How Does the Association of E7 With pRb Affect the Progression of Cancer?

pRb plays a role in cancer prevention by controlling the exit of cells from the cell cycle into G0/G1, thereby inhibiting cell cycle progression [49]. When in complex with the transcription factor E2F, pRb undergoes hyperphosphorylation, dissociating E2F from pRb [49]. E2F then binds to the promoters of various genes essential for DNA synthesis. However, E7 hinders the association of pRb with E2F by binding to pRb, leading to the continuous activation of E2F and the expression of genes involved in DNA synthesis (Figure 4) [43].

Using PM to Treat CC Through Inhibition of E6/E7

Numerous research studies have utilized the CRISPR-Cas9 mechanism to target the E6 and E7 oncogenes, ultimately impeding the progression of CC. By programming the guide RNAs to target exon segments of E6 and E7 specifically, CRISPR-Cas9 silencing inhibited growth, the cell cycle’s arrest, and cell death in cells infected with HPV 16 and 18 by restoring p53 and pRb. New and improved methods have also been discovered for effectively silencing E6/E7 expression, including zinc finger nucleases, and transcription activator-like effector nucleases [50]. In addition to adding E6 and E7 biomarkers to routine screening for CC, these personalized gene silencing mechanisms may contribute to the early intervention of CC, which will likely improve the effectiveness of treatment and increase an individual’s chances of survival. The E6/E7-inhibition example of the use of PM is outlined here highlighting the potential of such approaches in PPM in the near future, even at the stage of persistent HPV infection prior to the development of precancerous CIN. Such approaches will need development of ‘liquid biopsy’ biomarkers to identify appropriate patients (expanded further later on).

HPV35 and African descent

**Figure 5:**
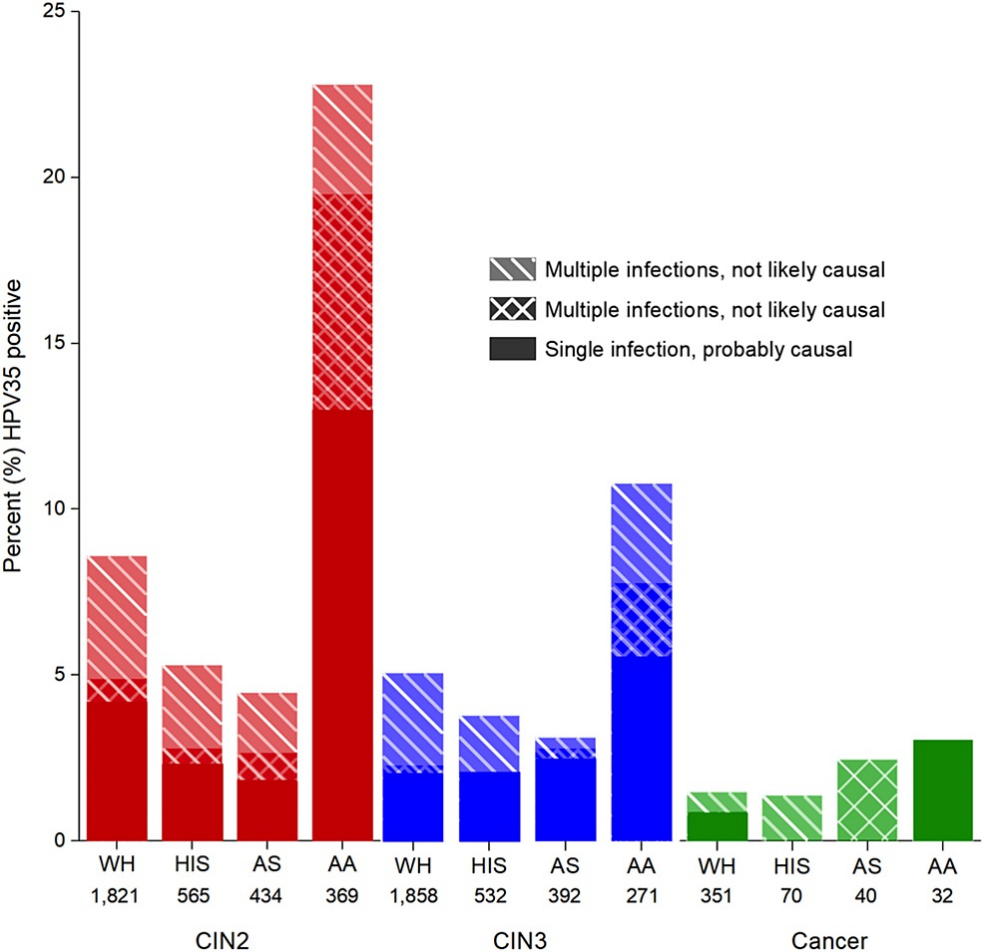
Prevalence of HPV35 among CIN2, CIN3, and cancer cases testing positive for carcinogenic HPV - identifying population-specific virus types is critical for tailored prevention. This image is reproduced from Pinheiro et al. [51], and permission was obtained from the licensed content publisher John Wiley and Sons. HPV, human papillomavirus; CIN2, cervical intraepithelial neoplasia grade 2; CIN3, cervical intraepithelial neoplasia grade 3; WH, White; HIS, Hispanic; AS, Asian; AA, African-American.

Although HPV is widely recognized as a leading cause of CC, the prevalence and risk for each variant differ, emphasizing the importance of identifying how specific types are incorporated into populations. Current PM covers several of the most prevalent variants of HPV, but recent studies have discovered the significance of HPV35 within sub-Saharan countries [51]. Studies noted that the HPV35 variant was responsible for only 2% of CC globally, but within the Sub-Saharan regions, it is associated with nearly 10% of invasive CC cases [51]. The nonvalent HPV vaccine (9vHPV), had a 90% estimated rate of preventing CC [52]. Interestingly, this specific vaccine in Sub-Saharan countries did not protect from HPV35-induced CC, the prevalent type in Africa when compared across the world [53]. African-American women were found to have higher levels of HPV35, and more HPV35-associated precancers compared to other ethnicities, so researchers conducted studies to identify the association between HPV35 infection and cervical pre-malignancies specific to women of different ethnicities. The results underlining the prevalence of HPV35 in African-American women with cervical intraepithelial neoplasia grade 2 (CIN2+), and cervical intraepithelial neoplasia grade 3 (CIN3+) are shown in Figure 5 [51].

CIN is characterized as a pathological state that precedes CC [54]. To inspect HPV35 sub-lineages, a whole-genome sequence was performed, resulted in finding two sub-lineages, A1 and A2, that were instrumental for comparing the association between HPV35 prevalence and cervical precancer/cancer for different ethnicities. Association between sub-lineages and ethnicities and their relation to increased prevalence of CIN3+ holds validity [51]. Evidence unveiled that African American women infected with the A2 sub-lineage and CIN3+ were positively associated while African American women with the A1 sub-lineage depicted an inverse association with CIN3+ [51]. Furthermore, a comparison of ethnicity-specific carcinogenicity between A1 and A2 variants has been explored. In general, women in the United States reported a greater frequency of infections leading to pre-malignancies by the A1 variant. In contrast, the Sub-Saharan population reported more cervical pre-malignancies induced by the A2 variant, represented in Table 2 [51].

**Table 2:** HPV sub-lineages provide specific targets for preventative medicine. This table is reproduced from Pinheiro et al. [51], and permission was obtained from he licensed content publisher John Wiley and Sons. HPV, human papillomavirus; HSIL, high-grade squamous intraepithelial lesion; CIN2, cervical intraepithelial neoplasia grade 2; CIN3, cervical intraepithelial neoplasia grade 3; ICC, invasive cervical cancers; n, number; %, percentage; CI, confidence limits; *P*-value, probability value; ^a^, Fishers' exact test; ^b^, Chi-square test.

Sublineages Regions	Benign infections			HSIL/CIN2/CIN3			ICC			P-value
	n	%	95% CI	n	%	95% CI	n	%	95% CI	
Non-Africa										
A1	93	94.9	88.6-97.8	15	93.8	71.7-99.7	43	89.6	77.8-95.5	
A2	5	5.1	2.2-11.4	1	6.3	0.3-28.3	5	10.4	4.5-22.2	.46^a^
Africa										
A1	62	45.9	37.7-54.3	8	57.1	32.6-78.6	18	58.1	40.8-73.6	
A2	73	54.1	45.7-62.3	6	42.9	21.4-67.4	13	41.9	26.4-59.2	.39^a^
P-value			<.0001^b^			.03^a^			<.01^b^	

The data suggest that the A2 variant has a more significant carcinogenic effect on the South African population than the A1 variant on women in the United States [55]. The evolution of the A2 sub-lineage to improve its fitness for the African ancestral host is discussed as a reason for these findings. Researchers also considered a bottleneck event that may have increased the resistance to infection by the A2 sub-lineage of HPV35 of non-African ethnicities [51]. Given that genomics is a significant driver of regional infection, race-specific vaccines, and screening could demonstrate potent efficacy. The removal of logistical barriers impeding the spread of vaccines and preventative medicine is another issue that must be resolved to see improvements in region-specific cancer prevalence.

Though CIN3+ is not a direct form of cancer, HPV causes CIN3+ in many cases, so CIN3+ is a principal indicator of a potential precancerous state [56]. HPV16 and 18 are known to be the most common types associated with CIN3+, cervical precancers, and CC when comparing all ethnic groups, but HPV16 is found to a lower extent when investigating the association of the variant with cervical precancers/cancers in people of African descent compared to other ethnicities [56-58]. Once again, this information supports the genotypic diversity of HPV types and how each variant affects different races to varying extents. Comparing HPV induced CC in Africa with regions of similar healthcare availability, HPV types specific to Mexico also show increased prevalence throughout the population. The leading HPV lineages and sub-lineages for CIN and CC have been identified for the Mexican population which further supports the urgency to incorporate population PM across the globe. By delving further into region specific HPV types and pathogenicity, preventative medicine can be customized for all populations [59]. With recent data providing a thorough insight into the association between HPV35 and CC in people of African descent, adding HPV35 to the current nonvalent HPV vaccine is of considerable value. As stated previously, the global HPV vaccine yields a prevention rate of 90% of CC and 80% of cervical precancer. This vaccination consists of nine HPV genotypes (HPV6, HPV11, HPV16, HPV18, HPV31, HVP33, HPV45, HPV52, and HPV58). The efficacy of the nonvalent vaccine is proven beneficial, but the impact HPV35 has on the Sub-Saharan population renders the equity of this vaccine questionable [56]. HPV16 is one of the most prevalent types in CC progression worldwide, so vaccination efforts are directed toward many strains, including HPV16. However, when Sub-Saharan regions are administered the commonly used HPV vaccines, precancer, and cancer risk increases because HPV35 and other non-vaccine HPV types are not being considered [57].

Given the effect that HPV35 has on the South African populations and relatives with shared genotypes, critical investigation of vaccinating African ancestral populations with HPV35 could potentially lead to a regression of CC rates in these regions. In addition to incorporating HPV35 into vaccines, screening for pre-malignancies associated with genotypic characteristics particular to Sub-Saharan regions encompasses the motives and mechanisms being pushed by PM. Observing these collective studies and further investigations involving the increased association of HPV35 and CC among people of African ancestry will hopefully provide insight into the future of population PM and how cancer prevention can be tailored to specific ethnic groups

Disproportionate impact on Hispanic women

Another population disproportionately affected by CC is Hispanic women. Compared to other groups, Hispanic women have a 32% higher incidence of CC than non-Hispanic white women and disproportionately high mortality rates compared to other ethnic groups [60]. Hispanic women are also more likely than many other groups to be diagnosed with an advanced stage of CC [61] and have been found to have the second-highest incidence of squamous cell CC as well as the highest incidence of both cervical adenocarcinoma and cervical adeno squamous carcinoma [62].

**Figure 6:**
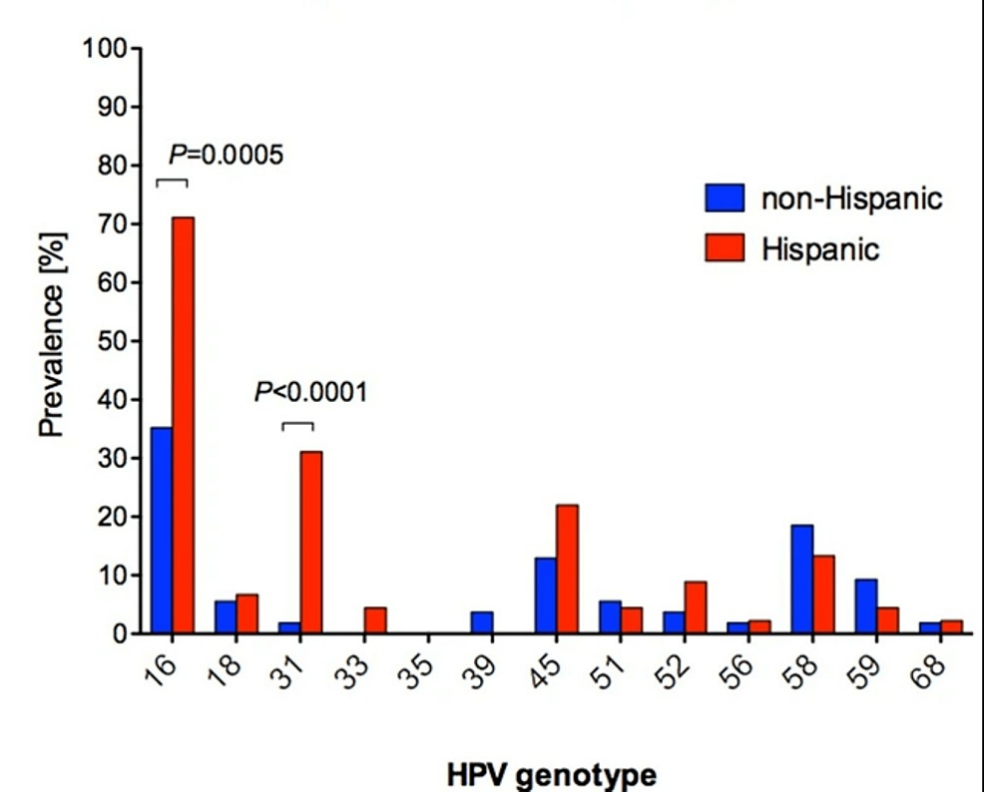
Common HPV genotypes in Hispanic women compared with non-Hispanic women. This image is reproduced from Lanieswski et al. [63], and permission was obtained from the licensed content publisher Springer Nature. P, probability; HPV, human papillomavirus; %, percentage.

Because of the causative effect of HPV on CC, it should continually remain a significant consideration in the most at-risk populations. However, the literature is varied concerning the most prevalent HPV types in the Hispanic population. As shown in Figure 6, HPV16 and HPV31 have been implicated with higher prevalence in Hispanic women than non-Hispanic women [63].

Because HPV16 is a genotype covered in the bivalent, quadrivalent, and nanovalent versions of the HPV vaccine, this finding could be misinterpreted as promising in protecting Hispanic women from HPV. However, other studies have found contradictory results, indicating that HPV16 may only be the seventh-most common genotype in Puerto Rican women [64] and that Hispanic women may be as little as half as likely to express prevalent HPV16 and HPV18, the only two genotypes targeted by all three of the major iterations of the HPV vaccine [65]. Perpetuated misunderstanding of these inconsistencies could be related to the increased relative prevalence of CC in the Hispanic population.

While many genotypes of HPV showed no significant difference in prevalence, HPV16 and HPV31 were more prevalent in Hispanic women compared with non-Hispanic women. In addition, while HPV31 is only targeted in the nanovalent HPV vaccine, HPV16 is one of two genotypes targeted in all three major vaccine iterations. This is a promising result for HPV prevention among Hispanic women but is inconsistent with data about vaccine efficacy among Hispanic women [63].

**Figure 7:**
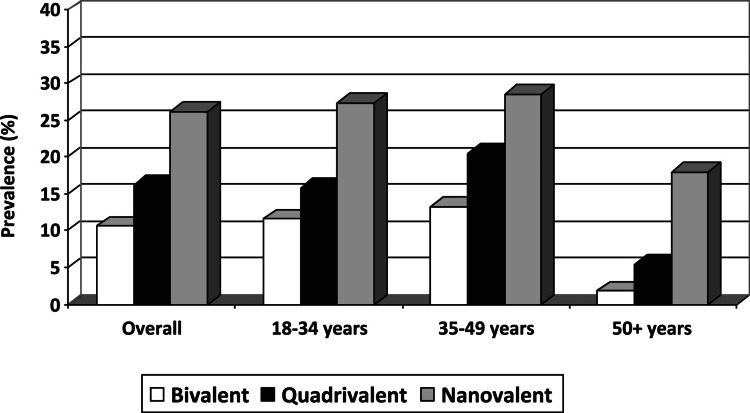
Relative prevalence of HPV genotypes targeted in the three major HPV vaccines among Puerto Rican women. This image is reproduced from Ortiz et al. [64], and permission was obtained from the licensed content publisher Elsevier. HPV, human papillomavirus; %, percentage.

Perhaps due to this inconclusive data, the currently licensed iterations of the HPV vaccine have consistently been found ineffective in preventing the HPV types most expressed in the Hispanic population. The previously mentioned Puerto Rico study found that a mere 10.6% of cases were positive for the HPV types in the bivalent vaccine (HPV16,18), 16.2% for the quadrivalent vaccine (HPV6,11,16,18), and 26.2% for the nanovalent vaccine (HPV6,11,16,18,31,33,45,52,58), which was consistent across age groups, as shown in Figure 7 [64].

Although each iteration of the HPV vaccine covers an increasing number of genotypes of the virus, none of the currently licensed vaccines adequately cover the genotypes present in Puerto Rican women. While subsequent iterations target increasingly more prevalent genotypes, even the nonvalent version does not reach 30% prevalence. This relationship was relatively consistent across age groups, with a drop off in prevalence for those 50 years of age or older [64].

Another study found that the prevalence of HPV genotypes covered by the more accessible bi- and quadrivalent vaccines was 20% in Hispanic women, second lowest to non-Hispanic black women. Hispanic women were also less likely to have nanovalent vaccine-covered genotypes (OR = 0.77). Overall, Hispanic women comprised the most significant proportion of those with HPV types not covered by any of the three major HPV vaccines [66]. Continued research is necessary in this area to confirm the most prevalent HPV genotypes among Hispanic women, continue the evaluation of vaccine efficacy, and consider potential alternatives for prevention specific to Hispanic populations.

Additional Targets for PPM Intervention

**Figure 8:**
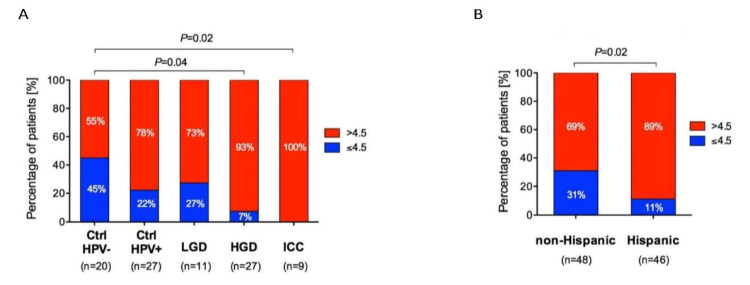
Vaginal pH in Hispanic and Non-Hispanic women associated with relative cervical cancer presentation and HPV prevalence. (A) Vaginal pH and severity of cervical neoplasm. (B) Vaginal pH and ethnicity. This image is reproduced from Laniewski et al. [63], and permission was obtained from the licensed content publisher Springer Nature. HPV, human papillomavirus; pH, potential of hydrogen; p, probability; LGD, low-grade cervical dysplasia; HGD, high-grade cervical dysplasia; ICC, invasive cervical carcinoma, n, number; %, percentage; -, negative; +, positive; >, greater than; < less than.

If inconsistencies in HPV genotyping and vaccinations continue, additional genetic factors and biomarkers could be considered targets for CC prevention in the Hispanic population. One study analyzing the genetic makeup of Hispanic and non-Hispanic CC patients found that 74 genes showed significantly higher rates of mutation among Hispanic patients than in the non-Hispanic group, resulting in significant alterations of the choline metabolism pathway in cancer, the Ras signaling pathway, and the primary pathways involved in cancer. One missense mutation in the PIK3CA gene (E545K) heavily involved with CC development and poor CC survival rates was also more prevalent in the Hispanic group than the non-Hispanic group [67]. Additionally, the cervicovaginal microenvironment is a potential area of exploration in the further characterization of CC. As shown in Figures 8A, 8B, Hispanic women have been found to have more diverse cervicovaginal microbiota, increased vaginal pH, and differential levels of specific species of bacteria, all correlated with cancerous growth severity and associated with the various presentations of CC [63].

In the above sample, Hispanic women had a significantly increased vaginal pH (B), which was both associated with a positive HPV sample and correlated with increasing severity of CC (A). Further research is needed to determine whether this is a potential CC prevention and management target or a byproduct of CC severity. Further research is necessary to reveal the relevance of these factors and the presence of others in the characterization, prevention, and treatment of CC among the Hispanic population.

Trends in CC Management in the Hispanic Population

Historically, a significant issue in the management of CC among Hispanic women has been a lack of screening due to accessibility and educational disconnects. Despite the increased CC prevalence among Hispanic populations, Hispanic women were more likely than other groups to have never received a Pap smear from 2004 to 2010. This effect correlated with increased age [68]. Fortunately, Medicaid expansions over the last fifteen to twenty years have made Pap smears and HPV vaccinations increasingly more accessible, dramatically improving screening rates among the Hispanic population. Low-income Hispanic women significantly benefitted, reporting the most significant increase across demographic groups and the largest Medicaid coverage increase [69].

However, the problems with education remain a troublesome obstacle in preventing CC among Hispanic women. Hispanic women have been reported to have the lowest CC knowledge score across demographic groups and the highest rate of agreement that their knowledge about CC was a barrier to their prevention and treatment [70]. A few suggested associations with the educational disparity are the language barrier and a cultural tendency towards fear and fatalistic beliefs concerning cancer, resulting in a lower desire to be aware of cancer, even if it is present [70]. However, no factor may be more significant than undocumented status. Compared to documented Hispanic women, undocumented Hispanic women have been reported to have lower knowledge about CC and experience more barriers to receiving an HPV test or a Pap smear.

Additionally, 88% of this population was uninsured, implying a financial barrier and lower access to the potential benefits of insurance expansion [71]. Fortunately, educational interventions concerning CC have boasted promising results, one example resulting in more excellent knowledge and more favorable attitudes about CC management among Hispanic women [72]. Hopefully, continued and widespread educational intervention about CC in concert with further understanding of its biological associations will reveal the aspects of CC prevention and management that Hispanic women can control and give them the knowledge and resources to do so.

Epigenetics

Epigenetic Modifications As Causative Factors of CC

**Figure 9:**
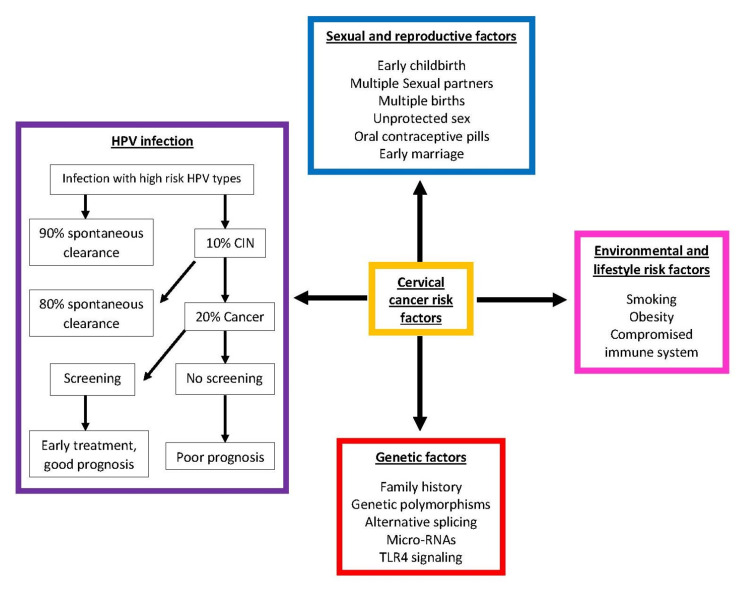
Risk factors for cervical cancer with a pathway for developing cervical cancer through HPV infection. This image is reproduced from Hull et al. [74] and is available via Creative Commons Attribution 4.0 International License. HPV, human papillomavirus, CIN, cervical intraepithelial neoplasia; RNA, ribonucleic acid; TLR, toll-like receptor.

Epigenetic changes play an extensive role in CC and may be utilized as an innovative prevention, diagnosis, and treatment tool. Epigenetics affects the expression of the human genome instead of altering the genome itself, categorizing these changes as separate from GM. As shown in Figure 9, several known risk factors, both biological and environmental, induce epigenetic changes in the human genome that cause CC [73,74].

Several epigenetic modifications arise from HPV infection and genetic, environmental, sexual, and reproductive risk factors contributing to CC development. While these epigenetic changes affect the expression of the human genome, HPV infection is associated with sexual and reproductive risk factors. Therefore, it is the most important risk factor in CC development.

These risk factors lead to multifactorial alterations in DNA methylation, miRNA expression, and histone modification. For instance, the genome accumulates DNA methylation in the known CC precursor and HPV-related cervical intraepithelial dysplasia [75]. This aberrant DNA methylation is strongly associated with tumorigenesis, mainly due to the dysregulation of oncogene and tumor suppressor gene expression [76]. In addition, varying epigenetic landscapes can induce either gene silencing or gene overexpression, resulting in an altered level of gene activity but no change to the actual genetic code. This review aims to highlight the interconnectedness of epigenetics as causative factors in CC and their utility in PPM.

Human behavioral and environmental factors also lead to epigenetic changes that promote cervical carcinogenesis. Nutrition, diet, alcohol use, smoking, and socioeconomic status have been found to all drive cancer-causing epigenetic changes [73]. For example, among several other dietary phytochemicals, curcumin (often found in turmeric) can inhibit HPV oncoproteins, slowing or preventing the growth of CC [77]. Inversely, alcohol intake lowers folate metabolism in CC through increasing DNA methylation and increased risk of HPV infection [78]. As such, among others, diets low in curcumin intake or high in alcohol consumption have been associated as causative determinants between risk factors and the onset of CC, and each is due to dysregulated epigenetic mechanisms and pathways. Unlike genetic alterations, epigenetic changes are reversible. Their reversible nature makes them susceptible to the effects of many different risk factors while suggesting their importance in future screening and therapeutics [73]. These dietary and lifestyle factors represent only two known environmental risk factors contributing to epigenetic modifications and CC.

CC Biomarkers and Screening

Researchers can use epigenetic alterations to prevent, detect, predict, and treat CC, including studying early DNA methylation modifications as observable and quantifiable biomarkers for CC [73]. DNA methylation affects genes related to the cell cycle and apoptotic pathways, DNA repair pathways, proliferation and growth regulation pathways, and invasion and metastatic pathways. Both hypomethylation and hypermethylation have been indicated as causative factors of CC, and each provides mechanisms for use as biomarkers of this disease [76]. In addition, although the mechanisms are still largely unknown, three specific microRNAs (miR-34a, miR-125, and miR-375) have been dysregulated in cervical exfoliated cells, demonstrating another potential molecular marker for CC [79]. Notably, these epigenetic changes have been well documented in their roles in cervical carcinogenesis; however, these changes are reversible, tissue-specific, and controlled by gene-environment interactions, which makes them desirable targets for the prevention, detection, and treatment of CC [73].

Using epigenetic biomarkers in CC recognition and therapy can increase prevention for populations. For example, 85% of CC deaths occur in developing regions, often due to a lack of access to healthcare [80]. Therefore, clinicians can focus on these populations to reduce CC rates through PPM. In addition, DNA methylation, miRNA alteration, and other epigenetic modifications can be detected noninvasively by sampling bodily fluids or exfoliated tissues [81]. Researchers are attempting to use these methods as markers for CC, which would serve as a tiny piece in the more significant role of PPM. Notably, serum miRNA (miR-638) combined with serum squamous cell carcinoma-related antigen has been shown to differentiate patients with cervical squamous cell carcinoma from healthy patients using qRT-PCR [82]. qRT-PCR is cost-effective but also convenient and accurate in screening techniques, allowing for population-wide testing. Further, liquid sample tests from exfoliative scrapings (i.e., QIAsure (Quiagen)) can be performed at home, thus affording sensitivity and privacy to patients; this increases access to CC detection in populations with already poor screening [75]. These studies effectively demonstrate CC screening strategies that can be employed on large populations more efficiently than traditional invasive measures, including cytology-based Papanicolaou (“Pap”) smears [82]. This literature demonstrates the power of liquid biopsy (blood or body fluid test) as a suitable detection tool for CC [75].

Epigenetic Changes and Therapeutics

Moreover, these same epigenetic changes can be reversed or inhibited with pharmaceutical treatments directly targeting the epigenetic change [83]. With an exhaustive list of epigenetic modifications, researchers are still determining novel therapeutics for each while repurposing current prescriptions for off-label use, including two common cardiovascular drugs and DNA methylation inhibitors, hydralazine and procainamide [84]. These studies suggest that PPM can be applied on a large scale to populations in developing regions with limited access to healthcare, mainly through epigenetic biomarker screening coinciding with novel and current pharmaceuticals as cost-effective, highly suitable tools against CC. Once pervasive risk factors have been identified in specific populations, and positive tests for serum or other fluid biomarkers have indicated an epigenetic modification, therapeutics could be employed to target that biomarker specifically for that population.

Three modalities of PPM in CC

How Can Other Modalities of PPM Improve CC Detection and Outcomes?

Clinicians can employ innovative PPM modalities to provide more efficient and accessible healthcare to targeted populations [8]. For example, the application of multi-cancer early detection (MCED) tests, big data, and telehealth each has the potential to improve access to healthcare for underserved populations through increased convenience and scalability [85-88] and, at times, cost-efficiency [85]. These three modalities and their role within CC detection and care are examined further in the following section.

Multi-cancer Early Detection: An Alternative Screening Method

MCED testing can conveniently reach in-need populations [85], mainly because MCED tests detect CC from a single blood sample and require fewer resources than traditional screening tests, including Pap tests, which usually require a physician office visit and cytology interpretation [73,85,89]. A lack of physicians, particularly in developing countries, leads to failed cytology-based screening program implementation. At the same time, other healthcare infrastructure issues create barriers to Pap tests, providing more reason to implement MCED testing [90]. However, although MCED tests provide an alternative screening technique, their cost-effectiveness still needs to be improved versus Pap tests, perhaps dissuading their widespread roll-out [91].

While traditional CC screening guidelines exist in several countries, more is needed to determine the utility of MCED as a potential CC screening measure in other populations [92]. Recently, Klein et al. determined that MCED tests maintain an 80% sensitivity and low false positive rate in predicting CC [93]. Hackshaw et al., however, determined varying screening performance based on the manufacturer, with overall cancer sensitivities ranging from 27% (CancerSEEK) to 100% (Pantum/EDIM) [85]. More randomized controlled trials would be valuable in determining the true efficacy of MCED, although MCED manufacturers currently present beneficial testimonials about their respective products. As risk factors vary for CC in different regions, evidenced, for example, by the lack of HPV vaccine coverage within the Hispanic population [66], MCED is even more valuable in low- and middle-income countries (LMIC) where CC rates are high and current practices are not sufficient [94]. MCED testing provides another possible avenue to increase CC detection, replacing traditional tests or adding to them in underserved populations.

Big Data Applications

Big data is defined as a massive amount of “smart” data from which unique insights can be made [95], and branches include machine learning and artificial intelligence, which may complement or challenge physicians’ roles in medicine [96]. The application of big data has the potential for increased CC prevention. For example, after a patient undergoes a Pap smear, automated histological image analysis has been sought to provide timely, accurate, and reliable results in the diagnosis and progression of CC sans physician [97]. In addition, automated image analysis may be employed in developing countries that lack trained clinicians, making time-efficient diagnoses and increasing CC screening participation. 

Although some Pap smear analysis algorithms have failed in countries that lack the funds for commercial software, other forms of big data applications have successfully detected CC [97]. For example, an innovative software named CervDetect uses machine learning algorithms with statistical analysis (i.e., Pearson correlation) to predict malignant cervical formation [86]. The results from this study showed 93.6% accuracy in predicting CC, which suggests CervDetect’s scalability to countries with elevated CC incidence [86]. Big data applications may provide direct preventative and diagnostic functions (i.e., CC prediction) and epidemiological trends and patterns. These analyses direct physicians’ and scientists’ efforts in understanding major risk factors and CC screening barriers unique to different populations and influencing strategies for lowering CC incidence in those regions.

Telehealth

Telehealth and other innovative healthcare tools have grown recently [98]. Since the beginning of the global COVID-19 outbreak in 2020, dramatic changes have occurred that have transformed the traditional practice of medicine (i.e., in-office visits) into more accessible medical support (i.e., video appointments or home screenings) [98,99]. Even before the pandemic, those in low- and middle-income countries (LMIC) have been predisposed to increased CC mortality due to low screening rates and diminished access to healthcare [87,100]. Still several studies have demonstrated the efficacy of telehealth and mobile phone usage in improving access to and increasing CC screening rates [101,102].

In Table 3, the example of African women receiving short message service (SMS) appointment reminders had 3.0 higher adjusted odds, while women receiving SMS messaging and an electronic eVoucher had 4.7 higher adjusted odds of attending a CC screening than the control group [101].

**Table 3:** Odds ratios for cervical cancer screening attendance in Africa by recipient group. This table is reproduced from Erwin et al. [101], and permission was obtained from the licensed content publisher BMJ Publishing Group Ltd. OR, odds ratio; SMS, short message service; eVocher, electronic vocher; CI, confidence limits.

Predictor	Crude OR	Adjusted OR	Adjusted OR 95% Wald CI	
SMS *vs. *control	3.31	3.04	1.49	6.21
SMS + eVocher *vs.* control	4.96	4.67	2.93	7.44
SMS + eVocher *vs.* SMS	1.50	1.53	1.11	2.19

Adjusted odds ratios of the SMS versus control group and SMS + eVoucher versus control group demonstrate the ability of telehealth messaging to increase CC screening participation among African women in combined urban and rural areas. Adding an eVoucher for roundtrip transportation to the screening further increased attendance rates.

This study demonstrates a beneficial outcome of telehealth on CC screening in regions with low screening rates [101], highlighting the capabilities of technological practices in increasing patient adherence to scheduled screenings and reaching more significant numbers of patients.

Self-sampling HPV tests are also an inexpensive alternative that can be mailed and performed at home instead of traditional cytology [102]. These shipped tests save women an office visit and an intimate cervical examination, allowing them to communicate with a physician remotely through a mobile device and an electronic health record [102]. This approach is cost-effective, time-efficient, and reliable while simultaneously highly scalable to LMICs that lack healthcare resources [102]. As a result, telehealth can reach greater populations of women, improve CC screening utilization, and lower CC incidence, especially in medically underserved areas and LMICs.

## Conclusions

PPM options such as E6/7-inhibition, the use of new biomarkers, the introduction of MCED tests, big data applications, and telehealth, detailed above, may increase access to CC detection and prevention through convenience, scalability, and cost-reduction, which perhaps would especially benefit low-and middle-income countries and other underserved populations. For example, women who might not have time to travel to distant healthcare facilities for CC screenings, particularly in rural areas, would benefit from these modalities/approaches and be more inclined to use them. Although further research remains on MCED, big data, and telehealth, each innovation provides a unique opportunity for PPM to expand access to CC-related healthcare in general and, more likely, to disadvantaged populations.

This review and perspective should be considered a primer and an introduction to the principles and use of PPM in CC. This report is meant for healthcare trainees of various disciplines and early career basic science researchers such as post-doctoral fellows eager to learn the potential clinical and translational interventions of new basic science inventions and discoveries in improving outcomes in CC. This field is rapidly changing. However, the authors hope the “big picture” outlined here about the potential of PPM and PPCM outlined here will stand the test of time and help eradicate CC at some point shortly. The authors hope this report will bring together various disciplines - basic scientists, clinical care providers, public health leaders, and population scientists to improve CC outcomes holistically and make further progress in the global elimination of CC.

## References

[1] Academy of Royal Medical Colleges (2024). The second report following the changing face of medicine national conference. https://www.thecfom.org.uk/_files/ugd/2b5a51_3c9fc8a3c2024bffa1d33d594e5238f4.pdf.

[2] Kulkova J, Kulkov I, Rohrbeck R (2023). Medicine of the future: how and who is going to treat us?. Futures.

[3] WP WP (2024). Changing the face of the healthcare. https://www.washingtonpost.com/sf/brand-connect/philips/wp/enterprise/changing-the-face-of-healthcare/.

[4] (2024). The changing face of medicine and the role of doctors in the future. https://www.bma.org.uk/media/2067/bma-the-changing-face-of-medicine-june-2017.pdf.

[5] Sutherland JM (2020). The changing face of healthcare delivery: making room for other disciplines. Healthc Policy.

[6] Patel MM, Adrada BE, Fowler AM, Rauch GM (2023). Molecular breast imaging and positron emission mammography. PET Clin.

[7] Bai JW, Qiu SQ, Zhang GJ (2023). Molecular and functional imaging in cancer-targeted therapy: current applications and future directions. Signal Transduct Target Ther.

[8] Vijayakumar S, Yang J, Nittala MR (2022). Changing role of PET/CT in cancer care with a focus on radiotherapy. Cureus.

[9] Schiavone F, Ferretti M (2021). The futures of healthcare. Futures.

[10] Barbazzeni B, Haider S, Friebe M (2022). Engaging through awareness: purpose-driven framework development to evaluate and develop future business strategies with exponential technologies toward healthcare democratization. Front Public Health.

[11] de Oliveira T, Tegally H (2023). Will climate change amplify epidemics and give rise to pandemics?. Science.

[12] Gustafsson LL (2023). Strengthening global health research. Glob Health Action.

[13] Baker RE, Mahmud AS, Miller IF (2022). Infectious disease in an era of global change. Nat Rev Microbiol.

[14] Gelband H, Jha P, Sankaranarayanan R, Gauvreau CL, Horton S (2015). Cancer: Disease Control Priorities.

[15] Mbogori T, Mucherah W (2019). Nutrition transition in Africa: consequences and opportunities. Global J Transformat Educ.

[16] Sung H, Siegel RL, Torre LA (2019). Global patterns in excess body weight and the associated cancer burden. CA Cancer J Clin.

[17] Zarocostas J (2022). The UN reports global asymmetries in population growth. Lancet.

[18] Hu B, Shi Y, Zhang P, Fan Y, Feng J, Hou L (2023). Global, regional, and national burdens of hypertensive heart disease from 1990 to 2019 ：A multilevel analysis based on the global burden of Disease Study 2019. Heliyon.

[19] Chetty R, Stepner M, Abraham S (2016). The Association Between Income and Life Expectancy in the United States, 2001-2014. JAMA.

[20] (2016). Global, regional, and national life expectancy, all-cause mortality, and cause-specific mortality for 249 causes of death, 1980-2015: a systematic analysis for the Global Burden of Disease Study 2015. Lancet.

[21] (2015). Global, regional, and national incidence, prevalence, and years lived with disability for 301 acute and chronic diseases and injuries in 188 countries, 1990-2013: a systematic analysis for the Global Burden of Disease Study 2013. Lancet.

[22] Hiatt RA, Beyeler N (2020). Cancer and climate change. Lancet Oncol.

[23] Vineis P, Huybrechts I, Millett C, Weiderpass E (2021). Climate change and cancer: converging policies. Mol Oncol.

[24] Ward MP, Malloy JS, Kannmacher C, Steinhubl SR (2023). Educating the healthcare workforce of the future: lessons learned from the development and implementation of a 'Wearables in Healthcare' course. NPJ Digit Med.

[25] Thibault GE (2020). The future of health professions education: Emerging trends in the United States. FASEB Bioadv.

[26] (2023). What is cancer?. https://www.cancer.gov/about-cancer/understanding/what-is-cancer.

[27] (2023). Evolution of a cancer (2016). Accessed: May 15. https://sphweb.bumc.bu.edu/otlt/mph-modules/ph/ph709_cancer/ph709_cancer5.html.

[28] Giroux V, Rustgi AK (2017). Metaplasia: tissue injury adaptation and a precursor to the dysplasia-cancer sequence. Nat Rev Cancer.

[29] Métayer J (1989). Gastric metaplasia and dysplasia. Relationship to cancer (Article in French). Acta Gastroenterol Belg.

[30] Tsikouras P, Zervoudis S, Manav B (2016). Cervical cancer: screening, diagnosis and staging. J BUON.

[31] (2023). What is cervical cancer?. https://www.cancer.gov/types/cervical.

[32] Ferrall L, Lin KY, Roden RB, Hung CF, Wu TC (2021). Cervical cancer immunotherapy: facts and hopes. Clin Cancer Res.

[33] Guo C, Qu X, Tang X, Song Y, Wang J, Hua K, Qiu J (2023). Spatiotemporally deciphering the mysterious mechanism of persistent HPV-induced malignant transition and immune remodelling from HPV-infected normal cervix, precancer to cervical cancer: integrating single-cell RNA-sequencing and spatial transcriptome. Clin Transl Med.

[34] (2023). Cervical cancer. https://www.who.int/news-room/fact-sheets/detail/cervical-cancer.

[35] (2023). United States Cancer Statistics: data visualizations. https://gis.cdc.gov/Cancer/USCS/.

[36] (2023). Mississippi cancer registry. https://www.cancer-rates.info/ms/.

[37] Tsimberidou AM, Fountzilas E, Nikanjam M, Kurzrock R (2020). Review of precision cancer medicine: evolution of the treatment paradigm. Cancer Treat Rev.

[38] Aftab M, Poojary SS, Seshan V (2021). Urine miRNA signature as a potential non-invasive diagnostic and prognostic biomarker in cervical cancer. Sci Rep.

[39] Baezconde-Garbanati L, Ochoa CY, Murphy ST (2020). Engaging Latinas in cervical cancer research. Advancing the Science of Cancer in Latinos.

[40] Bosch FX, Lorincz A, Muñoz N, Meijer CJ, Shah KV (2002). The causal relation between human papillomavirus and cervical cancer. J Clin Pathol.

[41] Tovar JM, Bazaldua OV, Vargas L, Reile E (2008). Human papillomavirus, cervical cancer, and the vaccines. Postgrad Med.

[42] Fowler JR, Maani EV, Dunton CJ, Gasalberti DP, Jack BW (2024). Cervical Cancer. https://pubmed.ncbi.nlm.nih.gov/28613745/.

[43] Pal A, Kundu R (2019). Human papillomavirus E6 and E7: the cervical cancer hallmarks and targets for therapy. Front Microbiol.

[44] Okunade KS (2020). Human papillomavirus and cervical cancer. J Obstet Gynaecol.

[45] Hu Z, Ma D (2018). The precision prevention and therapy of HPV-related cervical cancer: new concepts and clinical implications. Cancer Med.

[46] Tomaić V (2016). Functional roles of E6 and E7 oncoproteins in HPV-induced malignancies at diverse anatomical sites. Cancers (Basel).

[47] Zhou L, Qiu Q, Zhou Q (2022). Long-read sequencing unveils high-resolution HPV integration and its oncogenic progression in cervical cancer. Nat Commun.

[48] Somasundaram K (2000). Tumor suppressor p53: regulation and function. Front Biosci.

[49] Magaldi TG, Almstead LL, Bellone S, Prevatt EG, Santin AD, DiMaio D (2012). Primary human cervical carcinoma cells require human papillomavirus E6 and E7 expression for ongoing proliferation. Virology.

[50] Niebler M, Qian X, Höfler D (2013). Post-translational control of IL-1β via the human papillomavirus type 16 E6 oncoprotein: a novel mechanism of innate immune escape mediated by the E3-ubiquitin ligase E6-AP and p53. PLoS Pathog.

[51] Pinheiro M, Gage JC, Clifford GM (2020). Association of HPV35 with cervical carcinogenesis among women of African ancestry: evidence of viral-host interaction with implications for disease intervention. Int J Cancer.

[52] Mariani L, Preti M, Cristoforoni P, Stigliano CM, Perino A (2017). Overview of the benefits and potential issues of the nonavalent HPV vaccine. Int J Gynaecol Obstet.

[53] Okoye JO, Chukwukelu CF, Okekpa SI, Ogenyi SI, Onyekachi-Umah IN, Ngokere AA (2021). Racial disparities associated with the prevalence of vaccine and non-vaccine HPV types and multiple HPV infections between Asia and Africa: a systematic review and meta-analysis. Asian Pac J Cancer Prev.

[54] Katz IT, Butler LM, Crankshaw TL (2016). Cervical abnormalities in South African women living with HIV with high screening and referral rates. J Glob Oncol.

[55] Mix J, Saraiya M, Hallowell BD (2022). Cervical precancers and cancers attributed to HPV types by race and ethnicity: implications for vaccination, screening, and management. J Natl Cancer Inst.

[56] Huepenbecker SP, Meyer LA (2022). How can we pursue equity in cervical cancer prevention with existing HPV genotype differences?. J Natl Cancer Inst.

[57] Keller MJ, Burk RD, Massad LS (2018). Racial differences in human papilloma virus types amongst United States women with HIV and cervical precancer. AIDS.

[58] Ogembo RK, Gona PN, Seymour AJ, Park HS, Bain PA, Maranda L, Ogembo JG (2015). Prevalence of human papillomavirus genotypes among African women with normal cervical cytology and neoplasia: a systematic review and meta-analysis. PLoS One.

[59] Fragoso-Fonseca DE, Ruiz-Hernández UE, Trujillo-Salgado BB (2022). Analysis of the genomic diversity of human papillomavirus type 31 in cervical samples reveals the presence of novel sublineages in clade C. Arch Virol.

[60] Miller KD, Ortiz AP, Pinheiro PS (2021). Cancer statistics for the US Hispanic/Latino population, 2021. CA Cancer J Clin.

[61] Olusola P, Banerjee HN, Philley JV, Dasgupta S (2019). Human papilloma virus-associated cervical cancer and health disparities. Cells.

[62] Cohen CM, Wentzensen N, Castle PE, Schiffman M, Zuna R, Arend RC, Clarke MA (2023). Racial and ethnic disparities in cervical cancer incidence, survival, and mortality by histologic subtype. J Clin Oncol.

[63] Łaniewski P, Barnes D, Goulder A, Cui H, Roe DJ, Chase DM, Herbst-Kralovetz MM (2018). Linking cervicovaginal immune signatures, HPV and microbiota composition in cervical carcinogenesis in non-Hispanic and Hispanic women. Sci Rep.

[64] Ortiz AP, Tamayo V, Scorsone A (2017). Prevalence and correlates of cervical HPV infection in a clinic-based sample of HIV-positive Hispanic women. Papillomavirus Res.

[65] Montealegre JR, Peckham-Gregory EC, Marquez-Do D (2018). Racial/ethnic differences in HPV 16/18 genotypes and integration status among women with a history of cytological abnormalities. Gynecol Oncol.

[66] Montealegre JR, Varier I, Bracamontes CG (2019). Racial/ethnic variation in the prevalence of vaccine-related human papillomavirus genotypes. Ethn Health.

[67] Chandra S, Goswami A, Mandal P (2022). Molecular heterogeneity of cervical cancer among different ethnic/racial populations. J Racial Ethn Health Disparities.

[68] Chen HY, Kessler CL, Mori N, Chauhan SP (2012). Cervical cancer screening in the United States, 1993-2010: characteristics of women who are never screened. J Womens Health (Larchmt).

[69] Sabik LM, Tarazi WW, Hochhalter S, Dahman B, Bradley CJ (2018). Medicaid expansions and cervical cancer screening for low-income women. Health Serv Res.

[70] Akinlotan M, Bolin JN, Helduser J, Ojinnaka C, Lichorad A, McClellan D (2017). Cervical cancer screening barriers and risk factor knowledge among uninsured women. J Community Health.

[71] Mehta N, Raker C, Robison K (2021). Cervical cancer prevention: screening among undocumented hispanic women compared with documented hispanic women. J Low Genit Tract Dis.

[72] Valdez A, Napoles AM, Stewart SL, Garza A (2018). A randomized controlled trial of a cervical cancer education intervention for latinas delivered through interactive, multimedia kiosks. J Cancer Educ.

[73] Kabekkodu SP, Chakrabarty S, Ghosh S, Brand A, Satyamoorthy K (2017). Epigenomics, pharmacoepigenomics, and personalized medicine in cervical cancer. Public Health Genomics.

[74] Hull R, Mbele M, Makhafola T (2020). Cervical cancer in low and middle-income countries. Oncol Lett.

[75] Locke WJ, Guanzon D, Ma C, Liew YJ, Duesing KR, Fung KY, Ross JP (2019). DNA methylation cancer biomarkers: translation to the clinic. Front Genet.

[76] Bhat S, Kabekkodu SP, Noronha A, Satyamoorthy K (2016). Biological implications and therapeutic significance of DNA methylation regulated genes in cervical cancer. Biochimie.

[77] González-Vallinas M, González-Castejón M, Rodríguez-Casado A, de Molina AR (2013). Dietary phytochemicals in cancer prevention and therapy: a complementary approach with promising perspectives. Nutr Rev.

[78] Pathak S, Bhatla N, Singh N (2012). Cervical cancer pathogenesis is associated with one-carbon metabolism. Mol Cell Biochem.

[79] Pardini B, De Maria D, Francavilla A, Di Gaetano C, Ronco G, Naccarati A (2018). MicroRNAs as markers of progression in cervical cancer: a systematic review. BMC Cancer.

[80] Pimple S, Mishra G (2022). Cancer cervix: epidemiology and disease burden. Cytojournal.

[81] Bhat S, Kabekkodu SP, Varghese VK (2017). Aberrant gene-specific DNA methylation signature analysis in cervical cancer. Tumour Biol.

[82] Zheng S, Li R, Liang J (2020). Serum miR-638 combined with squamous cell carcinoma-related antigen as potential screening biomarkers for cervical squamous cell carcinoma. Genet Test Mol Biomarkers.

[83] Fang J, Zhang H, Jin S (2014). Epigenetics and cervical cancer: from pathogenesis to therapy. Tumour Biol.

[84] Dueñas-González A, Lizano M, Candelaria M, Cetina L, Arce C, Cervera E (2005). Epigenetics of cervical cancer. An overview and therapeutic perspectives. Mol Cancer.

[85] Hackshaw A, Clarke CA, Hartman AR (2022). New genomic technologies for multi-cancer early detection: rethinking the scope of cancer screening. Cancer Cell.

[86] Mehmood M, Rizwan M, Gregus Ml M, Abbas S (2021). Machine learning assisted cervical cancer detection. Front Public Health.

[87] Zhang D, Advani S, Waller J (2020). Mobile technologies and cervical cancer screening in low- and middle-income countries: a systematic review. JCO Glob Oncol.

[88] Uy C, Lopez J, Trinh-Shevrin C, Kwon SC, Sherman SE, Liang PS (2017). Text messaging interventions on cancer screening rates: a systematic review. J Med Internet Res.

[89] Pons-Belda OD, Fernandez-Uriarte A, Diamandis EP (2022). Multi cancer early detection by using circulating tumor DNA-the galleri test. Reply to Klein et al. The promise of multicancer early detection. Comment on "Pons-Belda et al. Can circulating tumor DNA support a successful screening test for early cancer detection? The grail paradigm. Diagnostics 2021, 11, 2171". Diagnostics (Basel).

[90] Catarino R, Petignat P, Dongui G, Vassilakos P (2015). Cervical cancer screening in developing countries at a crossroad: emerging technologies and policy choices. World J Clin Oncol.

[91] Deverka PA, Douglas MP, Phillips KA (2022). Multicancer screening tests: anticipating and addressing considerations for payer coverage and patient access. Health Aff (Millwood).

[92] Beer TM (2020). Novel blood-based early cancer detection: diagnostics in development. Am J Manag Care.

[93] Klein EA, Beer TM, Seiden M (2022). The promise of multicancer early detection. Comment on Pons-Belda et al. Can circulating tumor DNA support a successful screening test for early cancer detection? The grail paradigm. Diagnostics 2021, 11, 2171. Diagnostics (Basel).

[94] Hackshaw A, Cohen SS, Reichert H, Kansal AR, Chung KC, Ofman JJ (2021). Estimating the population health impact of a multi-cancer early detection genomic blood test to complement existing screening in the US and UK. Br J Cancer.

[95] Pastorino R, De Vito C, Migliara G, Glocker K, Binenbaum I, Ricciardi W, Boccia S (2019). Benefits and challenges of big data in healthcare: an overview of the European initiatives. Eur J Public Health.

[96] Johnson KB, Wei WQ, Weeraratne D (2021). Precision medicine, AI, and the future of personalized health care. Clin Transl Sci.

[97] William W, Ware A, Basaza-Ejiri AH, Obungoloch J (2018). A review of image analysis and machine learning techniques for automated cervical cancer screening from pap-smear images. Comput Methods Programs Biomed.

[98] Gorin SN, Jimbo M, Heizelman R, Harmes KM, Harper DM (2021). The future of cancer screening after COVID-19 may be at home. Cancer.

[99] Bartholomew K, Grant J, Maxwell A (2022). Feasibility and acceptability of telehealth and contactless delivery of human papillomavirus (HPV) self-testing for cervical screening with Māori and Pacific women in a COVID-19 outbreak in Aotearoa New Zealand. N Z Med J.

[100] Nnorom O, Sappong-Kumankumah A, Olaiya OR (2021). Afrocentric screening program for breast, colorectal, and cervical cancer among immigrant patients in Ontario. Can Fam Physician.

[101] Erwin E, Aronson KJ, Day A (2019). SMS behaviour change communication and eVoucher interventions to increase uptake of cervical cancer screening in the Kilimanjaro and Arusha regions of Tanzania: a randomised, double-blind, controlled trial of effectiveness. BMJ Innov.

[102] Woo YL, Gravitt P, Khor SK, Ng CW, Saville M (2021). Accelerating action on cervical screening in lower- and middle-income countries (LMICs) post COVID-19 era. Prev Med.

